# Parental conscription and cumulative adverse experiences in war-affected children and adolescents and their impact on mental health: a comment following Russia’s invasion of Ukraine in 2022

**DOI:** 10.1186/s13034-024-00732-0

**Published:** 2024-03-29

**Authors:** Katrin Erlewein, Emily Gossmann, Jörg M. Fegert

**Affiliations:** 1 Department of Child and Adolescent Psychiatry/Psychotherapy, University Hospital Ulm, Steinhövelstrasse 5, 89075 Ulm, Germany; 2 Competence Area Mental Health Prevention in the Competence Network Preventive Medicine Baden-Württemberg, Ulm, Germany; 3 German Center for Mental Health (DZPG), partner site Ulm, Ulm, Germany

**Keywords:** Children, Adolescents, War, Flight, Refuge, Parent-child separation, Conscription, Compulsory military service, Mental health

## Abstract

**Background:**

With Russia’s invasion of the Ukraine on February 2022, Ukrainian children and adolescents have been exposed to several stressful life events. In addition to the confrontation with war, flight and parent-child separation due to flight and forced displacement, the majority underwent another challenge at the initial phase of the war: the fatherly separation due to conscription.

**Main body:**

In the literature, the negative effects of exposure to war and flight/refuge, parent-child separation due to flight or forced displacement and parental deployment are well established. In the context of self-experienced war, the effects of parent-child separation caused by compulsory military service have not yet been sufficiently taken into account. However, the findings of the literature on the impact of these events on the mental health of children and adolescents show that they are at high risk for developing numerous psychological and behavioral problems.

**Conclusion:**

As children’s and adolescents’ mental health might be severely affected by war and its consequences, interventional programs that address the special needs of those children and adolescents are crucial.

## Background

Russia’s invasion of Ukraine on 24th February 2022 highlights again the several challenges war-affected children and adolescents are exposed to. Besides the ongoing threat to their lives, war-affected children and adolescents are also confronted with traumatizing experiences such as living in bunkers, forfeits in their education and health care, poverty and separation from their family, caregivers and other loved ones [1, 2]. But not only children and adolescents who remain in war-exposed countries experience these drastic events. People who seek refuge in general face most likely additional distress during their flight, too [2, 3]. This severely impacts children’s and adolescents’ daily lives and can also deteriorate their mental health.

The negative consequences of war, flight/refuge and parent-child separation due to flight and forced displacement are documented in empirical literature [4–13]. However, another reason for parent-child separation became apparent by Russia’s invasion of Ukraine: the compulsory military service of men [2]. The effects of a parent remaining in a war-torn country to serve in the war and the risk of being injured or killed on the mental health of children and adolescents have hardly been addressed in scientific research to date. However, this topic is highly relevant not only in the current context of Russia’s invasion of Ukraine but also because in some countries, laws still allow to enforce compulsory military service in times of war, crisis or national emergency [14] and new debates about reintroducing compulsory military service have arisen [14, 15]. While there is a large body of research on the psychosocial effects of parental voluntary military deployment on children and adolescents who remain in their safe home country (see Sect. 2.3.), research on the impact of parent-child separation due to conscription, especially in the context of self-experiencing war and children and adolescents themselves being forced to leave their home country is missing. This makes it difficult to draw conclusions on the psychological burden and the long-term effects on affected minors. Therefore, this comment aims to approach this topic by identifying the psychosocial effects of this multi-stressful situation.

## Main text

### Exposure to war and flight/refuge

Children and adolescents who are exposed to armed conflicts often experience several changes in their life simultaneously. This comprises direct consequences such as the risk of death, injuries, disabilities and torture [16] and indirect consequences as well [3, 16, 17]. The results of Betancourt and colleagues [18] illustrate that, among others, refugee children’s and adolescents’ exposure to war or political violence often co-occur with traumatic loss, forced displacement and community violence. Furthermore, the literature indicates that some refugee children and adolescents show signs of internalizing/emotional and/or externalizing/behavioral problems [18, 19], have a high probability of a clinically relevant disorder or psychological symptoms [18, 20] or other symptoms which “demand attention” [19] and/or fulfill the criteria for a mental disorder [18, 21]. Regression analyses show that together with other variables (e.g. community/motherly violence, engagement in coping strategies), exposure to war-related traumatic events predict Posttraumatic Stress Disorder (PTSD) symptoms, internalizing problems and prosocial behavior in refugee children and adolescents [22]. Lastly, the results of a meta-analysis reveal pooled estimates of 47% for PTSD, 43% of depressive symptoms indicative of depression and 27% of major anxiety disorder in children and adolescents affected by war [23].

The comparison of refugee/asylum-seeking children and adolescents to norms of typically developing children/normative values [24, 25], children who are from an ethnic minority but not refugees or indigenous white children [26] or children who have not experienced war and flight [27], partially reveal more emotional, behavioral and social problems as well as somatic complaints and impairments in cognitive development in refugee/asylum-seeking children and adolescents than in peers in the comparison groups [24–27].

### Parent-child separation due to flight or forced displacement

War, conflict and persecution are one of the most common reasons for parent-child separation [13] and parent-child separation due to war and flight is an adverse experience which can cause long lasting effects on children’s and adolescents’ mental health [28, 29]. Empirical studies show that unaccompanied refugee/asylum-seeking children and adolescents report having emotional, sleeping or (psycho)somatic problems [30] and score above ((borderline) clinical) cut-off values for various internalizing/emotional and/or externalizing/behavioral problems as well as psychological symptoms [30–32]. These results are confirmed by other studies which show that even after months and years after the separation some of the psychological symptoms and behavioral/externalizing problems remain the same [33–35]. Lastly, the results of Hampton and colleagues [36] demonstrate that in some forced family-separated children and adolescents mental and behavioral problems and psychiatric disorders may even persist after their reunification with their parents.

Regarding the comparison of unaccompanied asylum-seeking and refugee children and adolescents with accompanied asylum-seeking and refugee children and adolescents [37, 38] and/or native peers in the host country [39], some results indicate that unaccompanied asylum-seeking and refugee children are more likely to have posttraumatic stress symptoms, somatic complaints, emotional/internalizing and/or externalizing symptoms and a probable psychiatric diagnosis [37–39].

### Parental conscription and deployment

To our knowledge, there are currently no studies about the consequences of compulsory parental military service on the mental health of their offspring, only on their socioeconomic and behavioral outcomes [40–44]. However, the results drawn from literature regarding voluntary parental deployment show adverse outcomes for children’s and adolescents’ physical, psychosocial and economic well-being [45–54]. Literature regarding children’s and adolescents’ mental health shows overall adverse outcomes for children’s and adolescents’ internalizing and externalizing behavior and their mental health [49, 55–66]. Additionally, one study shows that extra health care visits of children and adolescents for mental health diagnoses are linked to parental deployment [67] and the length and the cumulative number of a parent’s deployment predicts increased psychological burden in children and adolescents that often remains even when the deployed parent returns home [49, 57, 60, 61, 67].

Comparative studies demonstrate that minors who experienced parental deployment have more mental health problems than those in civilian, national or community samples [49, 55, 58–61, 63, 67, 68]. However, studies comparing children of recently, currently or ever deployed parents with children of not or never deployed parents show mixed results [28, 57–59, 62, 69]. On the one hand, children of deployed parents show more mental health problems than children of non-deployed parents [28, 57, 58, 69]. On the other hand, there are studies showing no significant differences between the comparison groups but instead clinically significant symptoms in both groups [59, 62].

## Conclusion

Among the negative impact of exposure to war, fight and forced displacement as well as parent-child separation due to flight on children’s and adolescents’ mental health [see 13, 18–39], the adverse experience of parent-child separation due to the conscription of a parent may expose children and adolescents to another stressful situation that bears risks for serious impairments in their mental health. As shown by literature on deployment research, there is evidence that the fear of losing a parent due to serving in war leads to mental health problems in children and adolescents who are not themselves exposed to war [see 28, 49, 55–69]. Therefore, the same has to be assumed for more vulnerable children and adolescents, e.g. those, with their own experiences of war and forced displacement. Due to the circumstance that war, flight, forced displacement, parental separation, deployment and conscription may not occur individually but rather cumulate with one another and other additional adverse experiences and consequences [see 70–78], it becomes abundantly clear that tailored prevention and intervention programs for war-affected children and adolescents are urgently needed. The separation from a parent due to conscription and associated feelings should be addressed as a topic in prevention and intervention programs as well as in research. This applies not only to Russia’s ongoing invasion of Ukraine but also to all countries that currently face armed conflicts, some of which may not get a lot of media coverage (e.g. Ethiopia, Haiti, Myanmar, Sudan [79]) or those that will experience such unfortunate events in the future. It is also important to consider that in the case of Ukraine, Russia’s invasion started already in 2014 and an increase in mental health problems in war affected Ukrainian children and adolescents has already been observed before Russia’s invasion in 2022 [see 80]. Therefore, the adverse experiences that followed in the course of the current event (e.g. flight, parental separation) and the associated mental health problems in Ukrainian children and adolescents may have eventually cumulated with psychological distress that followed Russia’s previous invasion in 2014.

## Data Availability

No datasets were generated or analysed during the current study.

## Declarations

### Further information

A more detailed version of this commentary may appear in another journal at a later date.
